# Calcium phosphate cements for bone engineering and their biological properties

**DOI:** 10.1038/boneres.2017.56

**Published:** 2017-12-20

**Authors:** Hockin HK Xu, Ping Wang, Lin Wang, Chongyun Bao, Qianming Chen, Michael D Weir, Laurence C Chow, Liang Zhao, Xuedong Zhou, Mark A Reynolds

**Affiliations:** 1 Department of Endodontics, Periodontics and Prosthodontics, University of Maryland School of Dentistry, Baltimore, MD 21201, USA; 2 Center for Stem Cell Biology and Regenerative Medicine, University of Maryland School of Medicine, Baltimore, MD 21201, USA; 3 University of Maryland Marlene and Stewart Greenebaum Cancer Center, University of Maryland School of Medicine, Baltimore, MD 21201, USA; 4 Mechanical Engineering Department, University of Maryland Baltimore County, Baltimore, MD 21250, USA; 5 State Key Laboratory of Oral Diseases, West China Hospital of Stomatology, Sichuan University, Chengdu, Sichuan 610041, China; 6 VIP Integrated Department, Stomatological Hospital of Jilin University, Changchun, Jilin 130011, China; 7 Volpe Research Center, American Dental Association Foundation, National Institute of Standards & Technology, Gaithersburg, MD 20899, USA; 8 Department of Orthopaedic Surgery, Nanfang Hospital, Southern Medical University, Guangzhou, Guangdong 510515, China

## Abstract

Calcium phosphate cements (CPCs) are frequently used to repair bone defects. Since their discovery in the 1980s, extensive research has been conducted to improve their properties, and emerging evidence supports their increased application in bone tissue engineering. Much effort has been made to enhance the biological performance of CPCs, including their biocompatibility, osteoconductivity, osteoinductivity, biodegradability, bioactivity, and interactions with cells. This review article focuses on the major recent developments in CPCs, including 3D printing, injectability, stem cell delivery, growth factor and drug delivery, and pre-vascularization of CPC scaffolds via co-culture and tri-culture techniques to enhance angiogenesis and osteogenesis.

## Introduction

There has been a continuous and fast-paced emergence of new synthetic biomaterials developed for bone repair and regeneration over the past several decades. These biomaterials include metals, polymers, ceramics, bioactive glasses, calcium sulfates, calcium carbonates and calcium phosphates (CaPs). Among them, calcium phosphate cements (CPCs) are promising for clinical applications due to their advantageous properties including bioactivity, osteoconductivity, injectability and moldability. The discovery of the first CPC occurred inadvertently via the observation of calcium phosphate solubility behavior.^1–3^ Brown and Chow found that the solubilities of tetracalcium phosphate [TTCP: Ca_4_(PO_4_)_2_O], dicalcium phosphate (DCPA: CaHPO_4_) and dicalcium phosphate dehydrate (DCPD: CaHPO_4_ 2H_2_O) were much greater than that of hydroxyapatite (HA) under neutral pH conditions.^4^ A slurry containing appropriate amounts of TTCP and DCPD (or DCPA) led to HA precipitation as an end product and was capable of self-setting to form a hard mass.^2,3^ In the decade following this first discovery, CPCs were approved by the Food and Drug Administration (FDA) and were introduced into clinical practice for the treatment of craniofacial defects^5^ and bone fractures.^6^ Since then, other CPC formulations have been developed, and a large amount of research has been conducted.^7–18^ Currently, CPCs are defined as a combination of one or more calcium phosphate powders which, upon mixing with a liquid phase, form a paste able to self-set and harden *in situ* in the bone defect site to form a scaffold.^19^

**Figure 1:**
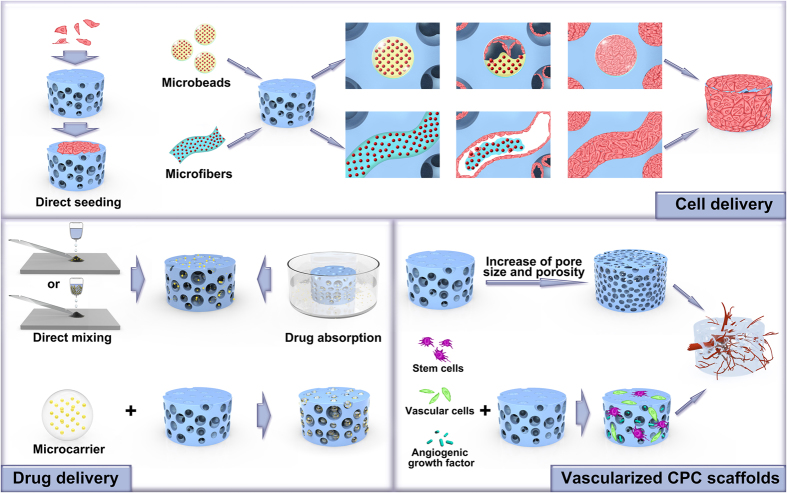
Schematic diagram summarizing the various CPC categories described in this article and their major biological properties.

One of the most important characteristics of CPCs is their ability to form *in situ* through a body-temperature dissolution-precipitation reaction.^19^ This feature gives rise to other beneficial properties such as molding capability upon mixing,^20^ injectability that enables minimally invasive application,^21^ and the ability to serve as a carrier for drug and biological molecule delivery.^22^ Early research on CPCs primarily focused on improved setting, handling and mechanical properties of CPCs through the tailoring of many processing parameters such as cement composition, additives, porogens, and particle size.^23–28^ In recent years, in addition to the development of new processing technologies in CPC manufacturing, the paradigm has shifted toward biological responses by emphasizing the enhancement of biological interactions of CPCs with cells and tissues as well as their applications in bone tissue engineering.^29–33^ Biological responses of scaffolds are a key factor in the translational application of biomaterials and their commercialization for clinic applications. Several meritorious reviews on CPCs have described their mechanical properties,^34–36^ processing approaches,^37,38^ drug delivery,^19,22,39,40^ and functional enhancement by polymeric additives,^41^ which will not be repeated here. The present article reviews the major new developments in CPC processing technologies in recent years and focuses on novel biological interactions of CPCs, particularly in the context of stem cell responses and delivery as well as *in vivo* bone regeneration. The various CPC categories described in this article and their major biological properties are summarized in the diagram in Figure 1.

## Pre-fabricated CPC scaffolds and 3D printing

Although injectability is one of the advantages of CPCs, pre-fabricated CPC scaffolds are often prepared for two reasons: (1) To ensure a complete setting reaction because only fully set CPCs demonstrate excellent tissue responses. When CPCs fail to set, they cause inflammatory reactions.^42^ Therefore, manufacturing pre-fabricated CPCs ensures complete setting prior to *in vivo* application. (2) To facilitate the creation of interconnected macroporous structures into CPCs. Self-setting CPC scaffolds without any modification are microporous but not macroporous and have limited pore interconnections.^43^ To promote tissue in-growth and accelerate the CPC degradation rate and subsequent replacement by bone, macropores were incorporated into CPCs via two methods: particle leaching (the addition of water-soluble particles, such as sodium bicarbonate, mannitol, salt or glucose, that dissolve or degrade after setting) and gas-foaming (the formation of air bubbles during the setting period).^37,44^ *In situ* setting with particle leaching has several disadvantages. First, because the porogens inside the cement have limited exposure to body fluids, the degradation or solubility of the particles may be compromised, which leads to limited porosity.^45^ Second, the *in vivo* dissolution of some particles may result in hyperosmosis.^46^ Third, some porogens may increase the paste viscosity and impede the injectability of CPC. The major drawback of *in situ* application of the gas-foaming method is the risk of air emboli or emphysema. Therefore, pre-fabricated CPC scaffolds have been developed to allow more delicate control of the setting process and macroporous architecture of the scaffolds before *in vivo* implantation.

Recently, three-dimensional (3D) printing has rapidly developed to allow the fabrication of pre-set CPC scaffolds. 3D printing is an additive manufacturing process in which geometrical data are used to produce 3D structures by depositing materials layer by layer.^47^ 3D-printed CPC scaffolds are favored over customization to meet the specific needs of each patient/defect. The benefits for clinical applications include easy adaptation and fixation, reduced surgical time, favorable esthetic results and minimal waste products. There are several different techniques for 3D printing, including direct 3D printing (direct ink writing), fused deposition modeling (FDM), stereolithography (SLA), and selective laser sintering (SLS). For a detailed description of each technique, readers are encouraged to read previous review papers on this topic.^48,49^ For CPC scaffolds, binder jetting is the most commonly employed 3D printing technique.^50^ Briefly, one or several print heads spray a binder solution (for example, an aqueous solution) precisely onto a bed layer of CPC powder. The binder locally joins adjacent powder particles together and hardens the wetted areas through the dissolution-precipitation reaction. The process repeats by spreading another layer of powder and ejecting binders according to a pass designed by the computer. This continues until the complete 3D structure is formed.^48^ The printability of the material is related to many parameters such as particle size and size distribution, morphology and surface area of the powder, roughness and flowability of the powders, the solubility/wettability/reactivity of the powder with the binder, and binder drop size.^51^ A study investigating beta-tricalcium phosphate powder suggested that 3D printing was not feasible with particles either too small (with a mean particle size of 7 μm) or too large (with a mean particle size of 51 μm), while mean particle sizes in the range of 20–35 μm resulted in good printing accuracy.^51^ Small particles tend to agglomerate under the influence of van der Waals forces. Very fine or porous particles exhibit low flowability and high surface roughness. Therefore, these factors greatly affect the smoothness and homogeneity of the powder bed, resulting in smearing and poor resolution.^51^ However, although large particles have better flowability, they tend to yield layer displacements due to low powder bed stability and low accuracy because the resolution is at least twice the particle size.^52^ Flowability was shown to be significantly reduced by decreasing the HA granule size.^53^ To work with small particle sizes to achieve a high resolution, strategies such as plasma coating^51^ and moisture application^54^ were attempted to stabilize the top layer surface and allow particle rearrangement and wetting while avoiding particle ejection out of the powder bed. Furthermore, by adding reactive minerals such as calcium sulfates into calcium phosphate, significant improvements to 3D printing parameters are achieved.^55^ The dimensional accuracy of printed CPC scaffolds (powder: alfa-TCP; liquid: Na_2_HPO_4_) is ~200·μm, which indicates a good degree of fitting to craniofacial defects in anatomical models.^56^ A critical step for powder-based 3D printing is the removal of the loose powder inside the pores of the printed scaffold after printing, a process known as depowdering. Depowdering is especially challenging when the pores and pore interconnections are small and found in the innermost parts of the scaffolds with large dimensions. One possible solution may be the use of depowdering-friendly designs with large windows and free-to-move fillers.^57^ In addition, layer thickness and printing orientations (parallel to the *X*, *Y* and *Z* directions) are important for depowdering.^58^ Shear forces at the powder bed increase with reduced layer thickness, which leads to the deterioration of the final printed samples upon depowdering. Depowdering is easier in scaffolds printed in the *X* and *Y* directions than that in scaffolds printed in the *Z* direction because of the distortion in samples printed in the *Z* direction.^58^ However, the relationship between 3D printing parameters and CPC scaffold quality and performance has yet to be established and warrants further study.

3D plotting (direct ink writing, direct write assembly, material extrusion) is another common technique for CPC 3D printing.^59^ This is an extrusion-based printing technology in which a paste or viscous materials, instead of powders, are used as the starting form and deposited as strands via a nozzle in a layer-by-layer fashion based on predesigned structures.^60^ For 3D plotting, the printability is dependent on even dispersion, viscosity, fluidity, extrusion performance, setting time of the paste, and the shape stability of the printed strands to withstand the weight of the structure during assembly. The setting time for CPCs plays an important role in controlling the printable time period of the paste. One study reported the printable time of a CPC (powder: TECP:DCPA=1:1 molar ratio, liquid/binder: polyvinyl alcohol) as only 10·min, which makes printing difficult.^61^ With the addition of a mesoporous calcium silicate, the printable time was increased to approximately 120·min.^61^ Other optimizations of the direct printing ink formulation have included the addition of gelatin to introduce an induction time for the onset of the CPC setting reaction.^62^ Specifically, this formula includes Targon 1128 as the dispersant, hydroxypropyl methylcellulose (HPMC) as the thickening agent, polyethylenimine (PEI) as the jellifying agent,^63^ and a ready-to-use oil-based CPC paste that sets only upon contact with water and thus has no time limit for printing.^59^

**Figure 2:**
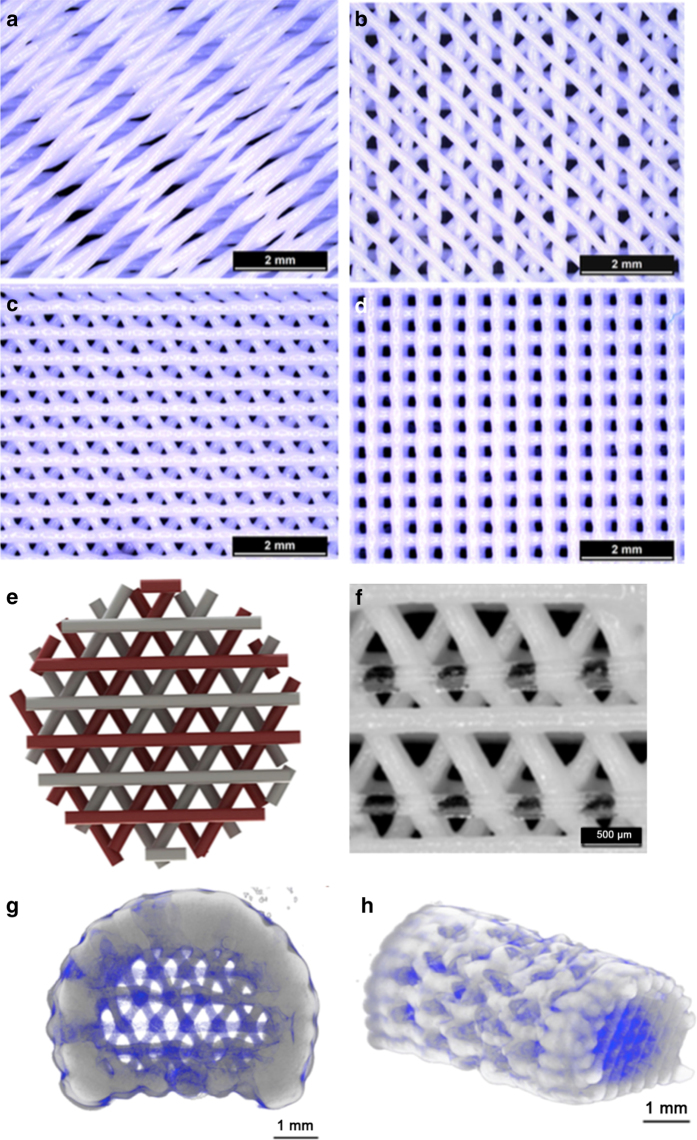
Highly sophisticated CPC scaffold structures via 3D plotting. Stereomicroscopic images of CPC scaffolds plotted with 15° (**a**), 45° (**b**), 60° (**c**) and 90° (**d**) configurations (change in orientation relative to the layer underneath). Design and printing of a CPC-hydrogel biphasic scaffold: model of biphasic scaffolds with CPC (white) and a growth factor-loaded hydrogel (red) (**e**); the printed scaffold (**f**); 3D reconstructions from micro-CT data of the biphasic scaffold (**g**,** h**). CPC is grayish white. Alginate-gellan hydrogel is blue. (Adapted from Ahlfeld *et al*.^64^ with permission.)

A critical issue for printing resolution is nozzle diameter and the stability of the extruded strands.^50^ 3D plotting has two advantages: (1) it enables easy printing of a combination of different materials,^64^ and (2) due to the mild conditions, it allows simultaneous cell or growth factor plotting, known as bioprinting.^64,65^ Using a two-channel plotting method, a scaffold with the combination of an oil-based CPC and an alginate-gellan hydrogel was fabricated and laden with growth factor VEGF, involving a highly sophisticated strand arrangement, pore structure and geometry (Figure 2).^64^ In another study, a bone morphogenetic protein 2 (BMP2)-loaded mesoporous silica/CPC porous scaffold was 3D-plotted and tested in *in vitro* cell culture and in a rabbit femur defect model.^66^ The scaffold promoted the osteogenic differentiation of human bone marrow stromal cells (hBMSCs) and enhanced vascularization and osteogenesis compared to the CPC control.^66^ In terms of cell-containing bioprinting, hydrogels such as alginate,^67^ collagen,^68^ synthetic polymers such as PLGA, and PEG^69^ are primarily used as bioinks due to their resemblance to the extracellular matrix (ECM) and good printability. In some cases, calcium phosphates are added to enhance cell attachment and osteogenic differentiation, thus favoring the use of bioink for bone tissue engineering applications.^67^

In general, due to the incremental addition of materials, 3D printing allows for not only the easy control of scaffold shape and geometry but also the control of fine features such as interconnected porosity, pore size and distribution, and complex spatial heterogeneity, which are not achievable with traditional strategies.^50^ The possibility of manufacturing customized implants with almost no design limitations makes 3D printing highly valuable in reconstructive surgery. However, more extensive research is needed to optimize the key parameters for successful 3D printing of CPC scaffolds.

## Injectable CPC scaffolds

Traditional bone grafting requires an open surgical approach to graft application sites and may be associated with complications such as a large surgical scar, increased pain and a longer post-operative recovery. To overcome these drawbacks, injectable bone graft substitutes are used for minimally invasive surgery. Two main obstacles that inhibit CPC injectability are liquid-solid phase separation during injection^70^ and paste disintegration upon contact with blood or body fluids.^71^ Phase separation leads to not only the presence of non-extrudable paste left in the syringe but also extravasation at the injection site and a decrease in the viscosity and mechanical strength of CPCs. The disintegration of CPCs in the body causes inflammatory responses and even severe consequences such as cement embolism and cardiovascular deterioration by simulating blood coagulation.^72^ Therefore, efforts have been made to improve CPC injectability. These strategies include the following: (1) increasing the viscosity of the liquid phase by adding viscous binders such as chitosan,^24^ gelatin,^73^ hyaluronic acid,^74^ methylcellulose,^75^ and others; (2) optimizing the CPC powder in terms of the particle size, particle size distribution, particle shape, and particle-particle interactions;^76^ (3) regulating the setting reaction;^77^ and (4) modifying the extrusion parameters such as CPC mixing and the sizes of the syringes and/or needles.^78^ All of these factors were discussed in detail in a recent review on CPC injectability.^70^

Recently, many studies have applied various injectable CPC formulations into animal models for bone regeneration.^79,80^ Injectable CPCs containing 50% (volume ratio) microspheres (poly(lactic-co-glycolic acid) (PLGA), gelatin (GEL) or poly(trimethylene carbonate) (PTMC)) were implanted into rabbit femoral bone defects. CPC/GEL had a significantly lower score than all other groups at the cement-bone interface. Both CPC and CPC/PLGA showed a better response than CPC/PTMC at 4 weeks, but there were no significant differences among these three groups at 8 and 12 weeks.^79^ A recent study applied a commercially injectable CPC (Calcibon) with platelet lysates in bilateral calvarial defects in rats.^81^ The delivery of the platelet lysate enhanced bone healing with an injectable CPC at early healing times. In large animal models, injectable CPCs have also shown promise for bone regeneration. For example, injectable CPC/PLGA composites demonstrated biocompatibility and direct bone contact for sinus floor augmentation procedures in a sheep model.^82^ Another study evaluated the efficiency of local bisphosphonate delivery via injectable CPC in vertebral bodies of the lumbar spine of an osteoporotic sheep model where the consequences of osteoporotic fractures were highly deleterious in patients. The bisphosphonate-combined cement in vertebral body bone defects had a beneficial impact on both bone content and the micro-architectural properties of the trabecular bone surrounding the implant.^83^ These animal studies demonstrated the promise of using injectable CPCs for bone repair and regeneration.

Indeed, CPCs have gained clinical acceptance as valuable bone substitution biomaterials for over 20 years, and several CPCs are commercially available. Injectable CPCs were used to repair human periodontal intrabony defects and showed favorable radiographic results.^84^ CPCs were also used in young patients for balloon kyphoplasty instead of polymethylmethacrylate cement. In most cases, good integration of CPCs in the vertebra was observed with no radiological signs of osteolysis or osteonecrosis. Only a few patients showed demineralization in follow-up CT scans.^85^ Several papers reviewing the properties of injectable CPCs are available for readers who want additional detail.^86–88^ The present review focuses on new developments in CPCs with an emphasis on their biological interactions and cell delivery as detailed in subsequent sections.

## Biological requirements and biological responses of CPCs

### Biocompatibility

Biocompatibility is defined as the property of a material being compatible with living tissues. Biocompatible materials do not induce a toxic response when implanted in the body.^89^ Biocompatibility is an essential requirement for tissue-engineered products to support cellular activities and optimize tissue regeneration without eliciting a cytotoxic effect in those cells or causing undesirable local or systemic responses in the host. The end products of the dissolution-precipitation reactions for CPCs include brushite (DCPD) and apatite (HA or calcium deficient HA (CDHA)), which are known to be biocompatible.^90^ Pre-set CPCs exhibit favorable short-term and long-term biocompatibility, as evidenced by many studies evaluating tissue responses in rats,^91,92^ rabbits,^93^ dogs,^94^ sheep,^16,32^ and goats,^95^ as well as various types of cultured cells.^24,93,96^ However, injectable CPCs require the completion of the setting reaction to avoid cytotoxicity, as unset or disintegrated CPCs cause severe inflammatory responses, blood clotting, and cement embolism.^72,97^ Incorporating polymers into CPCs is a strategy used to improve CPC properties.^41^ In a recent study, an injectable macroporous CPC was prepared by the syringe-foaming method using a hydrophilic viscous polymeric solution known as silanized-hydroxypropyl methylcellulose (Si-HPMC).^98^ Si-HPMC not only acts as a foaming agent to create macroporous structures inside CPCs but also endows the CPC paste with an appealing rheological behavior at the early stage of setting due to its self-crosslinking properties, thus improving its injectability and cohesion.^98^ Indeed, when this CPC was injected into defective rabbit femurs, no adverse foreign body reaction was observed at 1 week and 6 weeks post-implantation.^98^

### Bioactivity

Bioactivity refers to the ability of bone scaffolds to bind directly to the surrounding bone without the formation of fibrous tissue.^99^ Bioactivity is often evaluated by examining the ability to form apatite on the biomaterial in a simulated body fluid (SBF) with ion concentrations close to those in human blood plasma.^100^ A bioactive material is defined as one that accelerates apatite crystallization in a solution supersaturated with respect to hydroxyapatite.^100^ However, the validity of using an *in vitro* SBF test to predict the *in vivo* bioactivity of a material has been questioned.^101^ For example, Bohner and Lemaitre showed that a bioactivity test with SBF may not only give false-positive results but also false-negative results.^101^ The authors concluded that “*in vitro* bioactivity tests in SBF solutions cannot be used to predict the *in vivo* bone bonding ability of a material”. With some improvements to the protocol, these tests may be used for initial screening. However, the most reliable evaluation method remains *in vivo* implantation in a bone defect.

Bioactivity is one of the most important properties of CPCs.^19^ To further enhance CPC bioactivity, bioactive glass, which is known for its bioactivity, was incorporated into CPCs.^102,103^ The bioactive glass acted as a source of calcium and phosphate ions in the cement setting reaction. With this addition, increasing apatite formation was detected on the surface of the CaP compound after soaking in SBF for 7 days.^103^ *In vivo* examination of samples implanted into rabbit femoral bones indeed showed a better healing process and more bone growth with the addition of bioactive glass.^103^

### Osteoconductivity

Osteoconductivity is defined as a biomaterial property that facilitates the in-growth of new bone into a surface or a volume in which the biomaterial serves as a scaffold to guide new bone formation.^104^ CPCs are osteoconductive because they permit the attachment, proliferation, migration and phenotypic expression of bone cells, leading to the formation of new bone.^105,106^ Osteoconduction is related to the architectural geometry of the scaffold.^106^ Intimate adaptation, fixation and stability of the implant to the defect site are of critical importance to facilitate the in-growth of bone tissue. In addition, the scaffold should have high porosity and interconnectivity with optimal pore sizes to ensure cell penetration, nutrient exchange and waste elimination. For bone tissue engineering, an ideal scaffold should have 60%–80% interconnected porosity with pore sizes ranging from 150 to 500 μm.^107^

Osteoconduction also depends on the chemical composition of the scaffold. The incorporation of several types of ions benefit CPC osteoconductivity. For instance, a silicon CPC (Si-CPC) was developed,^108^ and the cytocompatibility of the Si-doped cement was tested with a human osteoblast-like cell line (MG-63), which showed enhanced cell proliferation (up to threefold) over that without Si. When implanted in a rabbit parietal bone defect model, significantly greater amounts of new bone were detected in the 10% Si-CPC group compared to that in the CPC control group.^108^ In another study, strontium was incorporated into CPC (Sr-CPC) to enhance its osteoconductivity and accelerate its degradation.^109^ *In vitro* studies showed higher osteoblastic cell proliferation rates in Sr-CPC groups. *In vivo* studies demonstrated more rapid degradation and advanced osteoconductivity in the 10% Sr-CPC group compared to those in the CPC control at 2, 4, 8, 16, and 32 weeks after the operation.^109^

### Osteoinductivity

Osteoinduction is defined as the recruitment and stimulation of progenitor cells to differentiate toward the osteoblastic lineage.^104^ CPCs are generally osteoconductive but not osteoinductive.^20^ However, several CPCs reportedly have the ability to form bone in nonosseous sites *in vivo* without the addition of osteogenic factors.^110^ Since this osteoinductive property is observed for some CPCs but not others, these materials are described as having “intrinsic” osteoinductivity.^111^ This inductive phenomenon is likely attributable to the combined effects of topography, composition, and micro and macroporosity of the CPC scaffolds.^111^ It is likely that the intricate architecture of the scaffold permits the entrapment and concentration of circulating growth factors, such as BMPs and osteoprogenitor cells, *in vivo* thus conferring osteoinduction capability upon the CPCs.^111^ In addition, CPCs serve as calcium and phosphate ion sources *in vivo*. Ca^2+^, PO_4_^3-^ and HPO_4_^2-^ ions are released into the surrounding tissues, regulate osteoblast functions^112^ and induce localized ion supersaturation, which causes the reprecipitation of carbonated apatite on the scaffold.^113,114^ A previous study proposed a new strategy to regulate bone marrow mesenchymal stem cell (BMSC) adhesion and osteogenic differentiation by adding magnesium into the CPC, thus improving its osteoinductivity.^115^ A CPC containing 5 wt% and 10 wt% magnesium not only enhanced BMSC adhesion but also upregulated osteogenic gene and protein expression *in vitro*. An *in vivo* study demonstrated that CPC with 5 wt% magnesium achieved the greatest bone volume at 2 and 8 weeks, confirming its beneficial osteogenesis effects via the addition of magnesium.^115^ To gain or enhance CPC osteoinductivity, novel strategies such as the addition of osteoprogenitor cells,^116,117^ growth factors,^118,119^ bioactive proteins^120,121^ or peptides^122,123^ into CPCs have exhibited favorable effects. Therefore, novel CPC compositions with intrinsic and engineered osteoinductivity are highly promising to enhance bone regeneration.

### Biodegradability

Ideally, a CPC scaffold should degrade at the same rate that new bone forms. CPCs biodegrade primarily via two mechanisms: a passive resorption process via chemical dissolution and an active resorption through a cell-mediated process.^124^ The degradation of CPCs is tailored by controlling several factors: (1) physical factors such as the physical form of the CPC (particulate or bulk), porosity, surface area, and crystallinity (crystal size, crystal perfection, and grain size), and so on; (2) chemical factors such as the composition and ionic substitutions; and (3) biological factors such as the activation of macrophages or osteoclasts.^125^ Enhancing CPC degradation is achieved by adding rapidly degradable porogens such as PLGA to generate macropores upon PLGA degradation. PLGA degrades hydrolytically, leading to the production of lactic and glycolic acid monomers. The acidic nature of the resulting byproducts is an additional advantage of PLGAs in combination with poorly degradable CPCs because CPCs degrade by acid dissolution.^126^ After being injected into a rabbit femoral bone defect model, CPC-PLGA exhibited favorable bone responses with >55% degradation and >13% bone formation at 6 weeks and >90% degradation and >40% bone formation at 26 weeks postoperation.^127^ Based on this same mechanism, glucono delta-lactone (GDL), which has a faster degradation rate than PLGA, was incorporated into CPCs as acid-producing microparticles to accelerate CPC degradation.^128^ Indeed, histomorphometrical evaluation revealed that CPCs containing 10% of GDL degraded more rapidly and were replaced by more bone tissue (32.8%) than CPC-PLGA at 2 weeks after implantation in a rabbit femoral bone defect.^128^

## CPC scaffold constructs for bone tissue engineering

### Cell delivery

Recent advancements in tissue engineering and regenerative medicine have indicated that cell-based therapeutics achieve robust regeneration with greater efficacy and better predictability than methods that do not involve cell seeding.^129^ These novel approaches employ scaffold constructs in combination with living cells to generate cell-driven, functional tissue rather than filling a defect with a nonliving scaffold. A tissue-engineered construct acts both as a scaffold to bridge the defect and as a cell delivery vehicle. The biomaterial-cell interactions of CPCs with various types of stem cells, such as BMSCs, umbilical cord mesenchymal stem cells (UCMSCs), embryonic stem cells (ESCs), induced pluripotent stem cells (iPSCs), were previously reviewed.^130,131^ The present article specifically explores recent advances in strategies for cell delivery, specifically highlighting the design of CPC-based scaffolds.

**Figure 3:**
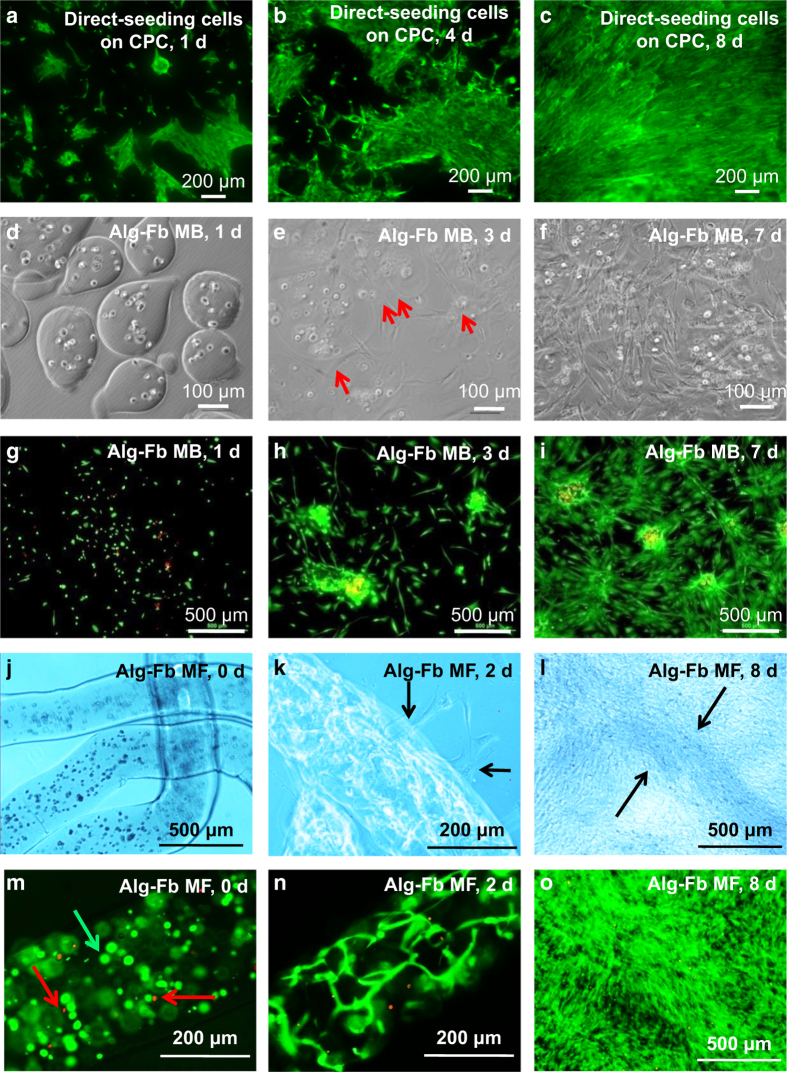
Methods of cell delivery via CPCs. Live-dead staining of (1) direct cell seeding on CPC surfaces (**a–c**); (2) cell encapsulation in alginate–fibrin microbeads (Alg-Fb MB) (**d-i**); (3) cell encapsulation in alginate–fibrin microfibers (Alg-Fb MaF) (**j–o**). (Adapted from Wang *et al*.^133,^ and Song *et al*.^139^ with permission.)

Direct cell seeding onto the porous surfaces of preformed CPC scaffolds is a common approach due to its simplicity. However, this type of static cell seeding has limitations, including low seeding efficiency and minimal cell penetration into the scaffold, leading to non-uniform cell distribution.^132^ It is not feasible to directly mix cells into the CPC paste because the mixing forces, ionic exchanges and pH fluctuation during CPC setting are detrimental to cell viability. To address this problem, cell encapsulation has been proposed to protect cells during CPC mixing and injection (Figure 3). In a recent study, human iPSC-derived MSCs (hiPSC-MSCs) were either pre-osteoinduced for 2 weeks (OS-hiPSC-MSCs) or transduced with BMP2 (BMP2-hiPSC-MSCs) to enhance their osteogenic capacity.^133^ The cells were then encapsulated in rapidly degradable alginate microbeads. The microbeads were mixed with CPC paste at a ratio of 1:1 and filled into cranial defects in nude rats.^133^ The results showed that the cells maintained good viability inside the microbeads after injection. Once the CPC set to form a scaffold, the cells were released as early as 3 days and demonstrated the up-regulation of osteogenic markers and bone mineral deposition. Cell-encapsulated groups produced greater amounts of new bone area *in vivo*, with 22.5%±7.6%, 38.9%±18.4%, and 44.7%±22.8% for the CPC-hiPSC-MSC, CPC-OS-hiPSC-MSC, and CPC-BMP2-hiPSC-MSC groups, respectively, compared to that for the non-cell CPC control group (15.6%±11.2%) at 12 weeks.^133^ Furthermore, the incorporation of cells accelerated the resorption of the CPC scaffold. The amount of residual CPC in the CPC-BMP2-hiPSC-MSC group was sevenfold less than that in the CPC control.^133^

Recently, rapidly degradable hydrogel fibers were developed for cell encapsulation and delivery.^134^ Encapsulation of cells inside microfibers possesses several advantages over microbeads. (1) Microfibers are easily fabricated by using a simple needle extrusion/external gelation method. To generate microbeads, air injection and electronic injection are needed to break up alginate droplets to form microbeads in sizes of several hundred microns.^135^ The air flow or electrostatic force during microbead formation may impose harsh shearing forces on the cells. Furthermore, the air flow forms “tails” on the microbeads, which may cause an immune response *in vivo*.^135^ (2) Microfibers with diameters of several hundred microns and millimeter-scale lengths are relatively easy to handle. (3) Microfibers provide more space for cellular self-assembly, through which living cells organize into functional units, allowing cells to grow, migrate and differentiate in the extracellular matrix.^136^ (4) Long microfibers form long macroporous channels with interconnectivity upon alginate degradation inside CPCs, while microbeads only form spherical pores with limited interconnectivity. These long channels improve osteoconductivity and nutrient and waste exchange of the scaffold. (5) Long microfibers potentially facilitate the formation of blood vessels in CPCs for bone engineering via co-seeding of endothelial cells and osteoblasts.

**Figure 4:**
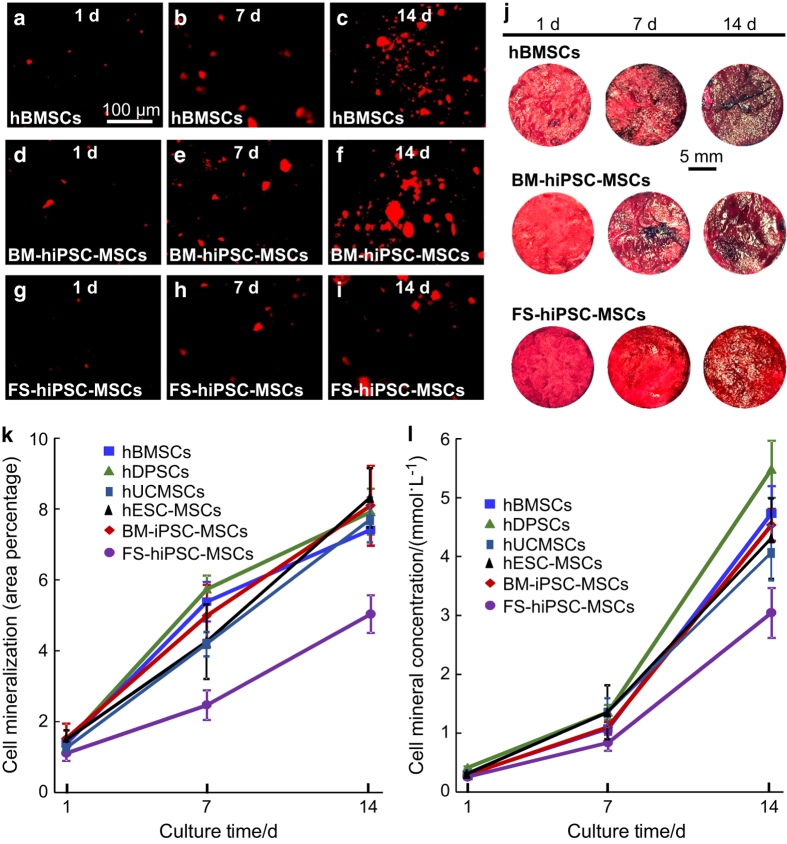
Synthesis of bone minerals by encapsulated stem cells. Images of (**a–c**) hBMSCs, (**d–f**) BM-hiPSC-MSCs, and (**g–i**) FS-hiPSC-MSCs stained with Xylenol orange (images of hESC-MSCs, hUCMSCs, and hDPSCs are similar to those of hBMSCs). (**j**) ARS staining of hBMSCs, BM-hiPSC-MSCs and FS-hiPSC-MSCs in CPC-CAF (images of hESC-MSCs, hUCMSCs, hDPSCs are similar to those of hBMSCs). (**k**) Xylenol orange mineral staining area (mean±s.d.; *n*=6). (**l**) ARS mineral concentration synthesized by cells in CPC-CAF (mean±s.d.; *n*=6). ARS: Alizarin red S, CAF: cell-encapsulating alginate–fibrin fibers. (Adapted from Wang *et al*.^137–^^138^ with permission.)

**Figure 5:**
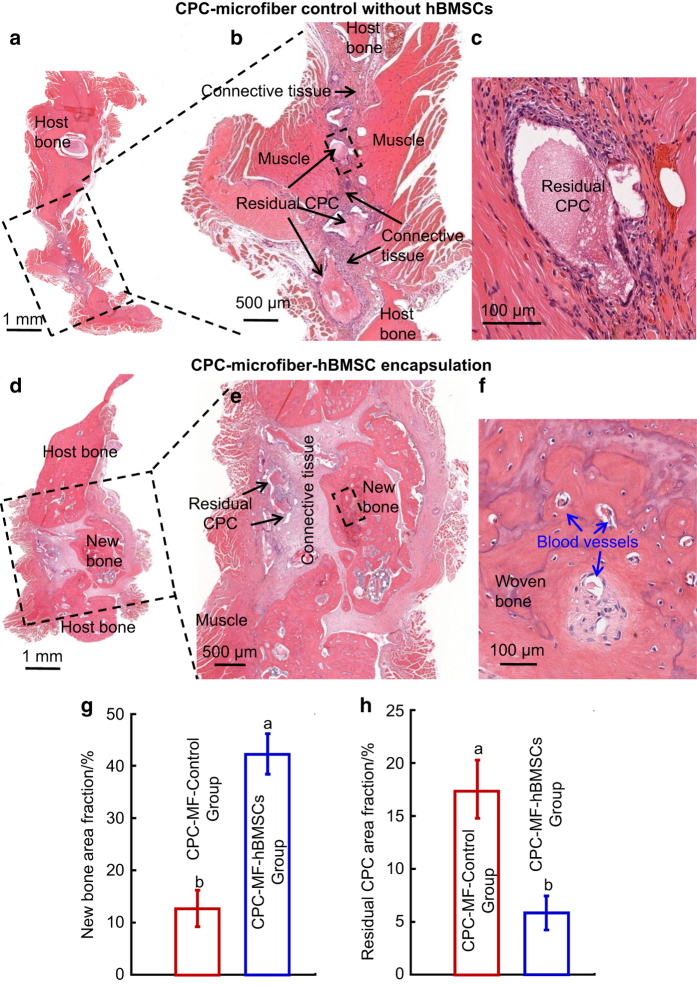
Representative h&e images at 12 weeks after surgery with the CPC-microfiber control group (**a**–**c**) and the CPC-microfiber-hBMSCs group (**d–f**) as well as quantification of the new bone area fraction (**g**) and residual CPC area fraction (**h**). Bone bridging was achieved in rat critical-sized mandibular defects in the CPC-microfiber-hBMSC group. The defect was closed with newly formed bone. (**b**) and (**c**), (**e**) and (**f**) are high-magnification images. Bars with dissimilar letters indicate significantly different values (*P*<0.05). Each value is the mean±SD (*n*=6). MF: microfibers. (Adapted from Song *et al*.^139^ with permission.)

Recent studies have encapsulated six types of stem cells, specifically hBMSCs, human dental pulp stem cells (hDPSCs), hUCMSCs, hESC-MSCs, and hiPSC-MSCs derived from bone marrow (BM-hiPSC-MSCs) and foreskin (FS-hiPSC-MSCs), in hydrogel microfibers and then delivered them inside an injectable CPC.^126,127^ The CPC paste encapsulating the stem cells was fully injectable under a small injection force, and the injection exerted no harmful effects on cell viability.^137^ The porosity of the microfiber-CPC construct was 62%.^138^ All six types of cells proliferated well and differentiated down the osteogenic lineage. hUCMSCs, hESC-MSCs, hDPSCs, BM-hiPSC-MSCs and hBMSCs exhibited high ALP, RUNX2, COL1A1, and OC gene expression. Cell-synthesized bone minerals increased with time, with no significant differences among hUCMSCs, hESC-MSCs, hDPSCs, BM-hiPSC-MSCs and hBMSCs, indicating good bone regeneration potential similar to gold-standard hBMSCs.^137,138^ However, FS-hiPSC-MSCs were inferior in terms of osteogenic differentiation compared to other cell types (Figure 4).^138^ In another *in vivo* study, an hBMSC-encapsulated microfiber-CPC paste was applied to repair rat cranial defects,^138^ and the hBMSC-encapsulated microfiber-CPC tissue engineering construct exhibited a robust capacity for bone regeneration. At 12 weeks, an osseous bridge in the rat mandibular defect was observed in the CPC-microfiber-hBMSCs group with a new bone area fraction of 42.1%±7.8%, which was threefold greater than that of the control group (Figure 5).^139^ Therefore, these results demonstrate that injectable hydrogel microfiber-CPC paste is a promising carrier for cell delivery and greatly enhances bone regeneration *in vivo*.

### Drug delivery

The non-exothermic setting reaction and the intrinsic porosity of CPCs allow the incorporation of drugs and biologically active molecules with low risk of thermal denaturalization or loss of activity during preparation or implantation.^19^ For drug incorporation into CPCs, the drug is simply mixed with either the liquid or solid components of the cement.^140^ Alternatively, it is added by adsorption onto the pre-set scaffold^141^ or incorporated into polymeric microspheres or microfibers before blending with CPC paste.^142^ Several factors influence the loading and release of therapeutic substances. These include the microstructure, porosity and surface area of the CPCs, the way in which the drug is incorporated into the CPCs, and the interaction between the drug and the CPC matrix.^19,143^ CPCs have been used as drug carriers for antibiotics^144^ as well as anti-cancer,^145^ anti-inflammatory,^146^ and anti-resorptive (anti-osteoporotic) drugs.^147^ CPCs have also been used as drug carriers for therapeutically active proteins or growth factors that foster local bone generation.^148^ Recently, ionically modified CPCs (for example, with Sr^2+^, SiO_4_^4−^, Zn^2+^, Mg^2+^) with the capability of influencing bone modeling and remodeling processes were investigated.^115,149,150^ For additional details, readers are referred to a review on the use of CPCs for drug delivery.^19^ Of note, the incorporation of the second phase of a degradable carrier into CPCs for drug delivery is beneficial for a more sustained release than directly loading the drugs into CPCs.^148^ For this purpose, gelatin microspheres,^151^ PLGA microparticles,^152^ bioactive glass,^148^ and chitosan/dextran sulfate microparticles^153^ have been used in CPCs to deliver drugs with tailored degradation rates to control the release profiles.

### Vascularized CPC scaffolds

Adequate and rapid vascularization is essential for successful bone regeneration. Failure of the bone healing process, including delayed healing or non-unions, is often attributable to a lack of adequate vascularization.^154^ Furthermore, vascularization is critical for the viability of seeded cells in the scaffold. If the distance between cells and the nearest capillary network is greater than 100–200·μm, which exceeds the diffusion or perfusion limits of nutrients and oxygen, the viability of the seeded cells is compromised.^89^

Improvement in CPC vascularization is stimulated by modifications to the material itself. Physical features such as porosity and pore sizes are known to impact vascularization.^155,156^ To this end, a study fabricated a self-setting CPC composite with gelatin fibers to create interconnected hollow channels in the CPC after dissolution of the gelatin fibers.^157^ *In vivo* subcutaneous implantation showed that the resulting channels in CPC indeed facilitated vascular infiltration into the construct.^157^ In addition, different channel sizes induced different vascularization behaviors *in vivo*. Channels with a 250-μm diameter increased the expression of the representative angiogenic factors HIF1α, PLGF and migration factor CXCR4, which induce the formation of small vessels. Channels with a larger diameter of 500 μm enhanced VEGF expression, which induces the development of large vessels. More HIF1α-positive cells were found in the interconnected intersections of several channels, indicating high levels of sprouting and vasculogenesis potential under hypoxic conditions.^157^ While the majority of research has focused on modifying the physical features of CPCs to improve vascularization, chemical features, such as the release of ionic calcium and phosphate, have also been suggested to play a role in regulating vascularization.^158^ In a recent study, CPCs were coated with a graphene oxide-copper nanocomposite with the rationale that the oxygen-containing functional groups in graphene oxide would provide more binding sites for serum proteins and thereby enhance initial cell adhesion and other bioactivities.^159^ When incubated with rat BMSCs, CPCs with the novel graphene oxide-copper nanocomposite coating activated Hif-1α and further enhanced the expression of VEGF and BMP-2 via the Erk1/2 signaling pathway. Indeed, an *in vivo* study found more blood vessel volume and bone regeneration in the coated-CPC group.^159^ However, the mechanism underlying vascularization and the impact on bone regeneration efficacy via CPCs require additional experiments, particularly *in vivo* studies.

**Figure 6:**
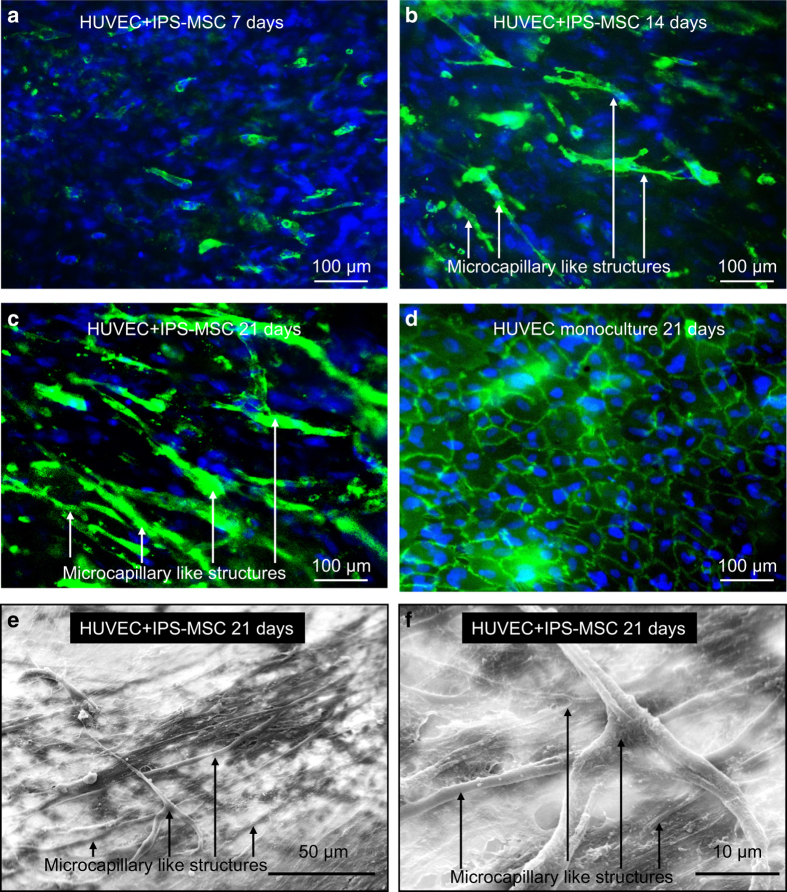
Formation of microcapillary-like structures by HUVECs and hiPSC-MSCs co-cultured on CPC scaffolds at 21 days (**a**-**c**). HUVECs were identified by immunostaining with the endothelial marker PECAM1 in green on the cell membrane, and nuclei were stained with DAPI in blue. hiPSC-MSCs were identified by nuclei counterstained with DAPI in blue but lacking green staining on the cell membrane. Microcapillary-like structures increased with culture time. d shows the HUVEC monoculture control group, which exhibited no evidence of vascular-like structures. Representative SEM images of microcapillary-like structures via the co-culture system (**e**,**f**). (**f**) A higher magnification image of the image in **e**. (Adapted from Liu *et al*.^165^ with permission.)

From a biological point of view, angiogenic growth factors, stem cells and vessel-forming cells are highly promising approaches to promote vascularization. A recent study investigated the use of autologous BMSCs in combination with autologous platelet-rich plasma (PRP) delivered via a macroporous CPC to regenerate large bone defects in minipigs.^160^ The CPC-BMSC-PRP group generated twofold more new bone and twofold higher blood vessel density compared to those of the macroporous CPC control at 12 weeks.^160^ In addition, recombinant growth factors and cell signaling molecules are alternatives to autologous growth factors that provide more flexible and delicate control over the dose and factors to be incorporated. Several studies have loaded dual agents, specifically BMPs and VEGF, in a single CPC scaffold, which demonstrated excellent angiogenic activity *in vitro* and *in vivo*.^161,162^ In addition to using growth factors, CPC pre-vascularization *in vitro* was investigated.^163^ In this method, vessel-forming cells were co-seeded with bone-forming cells on the engineered tissue construct to form microvascular structures before implantation *in vivo*. The co-culture of human osteoblasts and human umbilical vein endothelial cells (HUVECs) on gas-foaming macroporous CPCs *in vitro* successfully generated microcapillary-like structures and elevated the expression of angiogenic and osteogenic markers.^163^ Furthermore, the beneficial effects of co-culture were amplified by using an Arg–Gly–Asp (RGD) modification for the CPC scaffold.^164^ Similarly, the co-culture of hiPSC-MSCs and HUVECs on a macroporous CPC *in vitro* also generated microcapillary-like structures (Figure 6).^165^ In an animal study, HUVECs were co-cultured with four types of stem cells, specifically hUCMSCs, hBMSCs, hiPSC-MSCs and hESC-MSCs, on CPCs and then implanted in an 8-mm critical cranial bone defect in rats for 12 weeks.^166^ Microcapillary-like structures were successfully formed on CPCs *in vitro* in all four co-culture groups. New bone formation and the blood vessel densities of the co-cultured groups *in vivo* were much greater than that of the CPC control without cell seeding or the CPC-BMSCs group without co-culture (*P*<0.05).^166^ These results demonstrated the promise of co-culture and CPC pre-vascularization to greatly enhance osteogenesis and angiogenesis *in vivo*.

**Figure 7:**
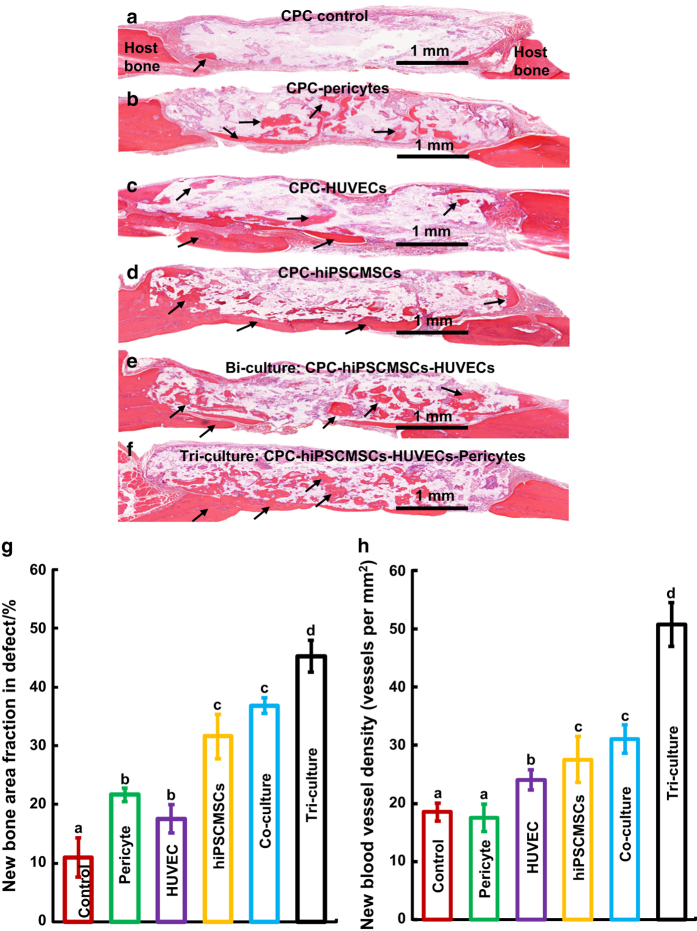
Representative h&e images at 12 weeks after the implantation of CPC scaffolds generated utilizing different pre-vascularization strategies in rat cranial bone defects. Mineralized new bone is stained in red (black arrows). The white area is attributable to slight detachment of the tissue. The dura is at the bottom. Cell-seeded groups had more new bone than the CPC control. Much higher amounts of new bone formed in the tri-culture group. Histomorphometric analysis of the fraction of new bone (g) and new blood vessel density (h). The tri-culture group had the greatest amount of new bone and new blood vessel density among all groups (*P*<0.05). Each value represents the mean±sd (*n*=6). Dissimilar letters indicated significantly different values (*P*<0.05). (Adapted from Zhang *et al*.^169^ with permission.)

For successful bone regeneration, it is important to establish vascularization in a timely manner, but the stabilization of such a vascular network is of similar importance, although it is often neglected. Angiogenesis without vessel maturation produces abnormal, defective blood vessels that are prone to regression.^167^ Perivascular cells such as pericytes play important roles in the stabilization and maturation of blood vessels by guiding the developing vessels to respond to angiogenic stimuli.^168^ Enlightened by this fact, further improvement of the pre-vascularization strategy with the addition of pericytes was attempted.^169^ A tri-culture system comprising hiPSC-MSCs, HUVECs and pericytes was developed to pre-vascularize the CPC scaffolds.^169^ Both the bi-culture and tri-culture groups exhibited the formation of vessel-like structures *in vitro*, greatly elevated levels of angiogenic and osteogenic markers, and bone matrix mineralization. After implantation in a rat model with a cranial bone defect for 12 weeks, the tri-culture group demonstrated much higher amounts of new bone than the bi-culture and monoculture groups and the CPC control (Figure 7).^169^ The substantial increase in bone formation in the tri-culture group was likely related to enhanced vascularization and the stabilization and maturation of blood vessels.

*In vivo* pre-vascularization is also achieved using a surgical method involving the implantation of a scaffold into a well-vascularized and easily accessible body tissue such as a subcutaneous pocket or a muscle pouch. Microvascular structures are formed as a result of invasion and outgrowth of the surrounding host microvasculature.^170,171^ After the completion of pre-vascularization, the tissue construct is harvested and grafted into the defect site, where the preformed microvessels inside the construct inosculate and anastomose with the host blood vessels. The disadvantages of this approach are obvious: the invasive nature of the surgery, higher cost, and a relatively longer treatment process. Therefore, new tissue engineering methods utilizing CPC scaffolds with co-culture and tri-culture represent exciting alternative strategies that warrant further research for continued improvement to achieve wide clinical applications.

## Conclusions

Due to their injectability, bioactivity and biocompatibility, CPCs are highly promising for bone tissue engineering applications and are used as scaffolds and carriers to deliver stem cells, drugs and growth factors. CPCs are either used as pre-set scaffolds or injectable pastes. 3D printing is a promising technology for fabricating CPC scaffolds with a high degree of accuracy and is used to develop intricately detailed biomimetic structures that are not achievable via traditional manufacturing methods. 3D printing has the potential to facilitate the next generation of smart and functional CPCs. Furthermore, with recent advances in tissue engineering, a new emphasis on “tissue regeneration by natural tissues” instead of “tissue replacement by biomaterials” has been proposed. Thus, CPCs with excellent biological interactions, such as osteoconductivity, osteoinductivity, biodegradability and bioactivity, are promising to meet this need. CPC composite constructs and hybrid systems involving the incorporation of cells, growth factors, bioactive molecules, bioinorganics, polymers, and bioactive glass are likely to yield favorable bone regenerative outcomes and greatly widen the clinical applications of CPCs. In addition, the co-culture and tri-culture of various tailored cell types with CPC scaffolds offer exciting potential for vascularization in bone tissue regeneration, which is especially important for treating large-sized bone defects. Further studies are needed to realize these promises and understand the underlying mechanisms to further the development of tissue engineering and regenerative medicine.
